# Textural, Color, and Sensory Analysis of Cookies Prepared with Hemp Oil-Based Oleogels

**DOI:** 10.3390/gels11010046

**Published:** 2025-01-07

**Authors:** Ana Leahu, Cristina Ghinea, Sorina Ropciuc, Cristina Damian

**Affiliations:** Faculty of Food Engineering, Stefan cel Mare University of Suceava, 720229 Suceava, Romania; analeahu@fia.usv.ro (A.L.); sorina.ropciuc@fia.usv.ro (S.R.); cristinadamian@fia.usv.ro (C.D.)

**Keywords:** dough, fat substitution, oleogel, hemp seed oil, rheology, texture analysis

## Abstract

The amount of saturated fat in cookies can be reduced by replacing margarine with oleogel, resulting in healthier products. In this study, the rheological and textural profile of cookies formulated with oleogel as the main margarine substitute was evaluated. Hemp seed vegetable oil was oleogelized with four types of waxes: beeswax (BW), carnauba wax (CW), candelilla wax (DW), rice bran wax (RW), and three oleogeling agents, sitosterol (S), pea protein (PP), and xanthan gum (XG), respectively. The textural and rheological properties of the oleogel dough samples were analyzed using the PertenTVT-6700 texturometer (Perten Instruments, Sweden) and the Haake rheometer. The results showed an increase in the hardness of cookie doughs with oleogels. The values of the elastic component (G′) and the viscous component (G″) increased, which means that the oleogels used affected the rheological behavior at 25 °C, causing an increase in the dough consistency. Sensory attributes, texture, and color parameters of cookies with oleogels were determined. The cookies’ hardness increased significantly from 4409.83 ± 0.13 g (control sample) to 7085.33 ± 0.15 g in the cookie sample prepared with hemp oil sitosterol oleogel, whereas the sample with candelilla wax had the lowest hardness value of 4048.09 ± 0.14 g. The color of the oleogel cookies was darker than that of the control cookies. The cookie sample with hemp oil and beeswax oleogel was the most appreciated by the evaluators among the oleogel cookie samples. The findings suggest that hemp seed oil oleogel is an effective fat substitute in cookies, promoting the application of this vegetable oil in food products.

## 1. Introduction

The globalization of markets has transformed food systems and led to major changes in food consumption behavior (such as replacing family meals with popular fast foods and reducing cooking time), which can be influenced by concern for the health and nutritional status of the population. High-fat snacks, like cookies, are very popular among consumers. According to Drewnowski and Almiron-Roig [1], fats contribute to the texture, flavor, and aroma of food products. The consumption of high-fat snacks (containing trans and saturated fatty acids and sugar) has been linked to an increased risk of obesity [2], diabetes [3], colorectal cancer [4], and cardiovascular disease [5]. Childhood obesity is the most common chronic disease in children and adolescents, and changes in eating and physical activity patterns have led to a 30% higher childhood obesity rate in developing countries than in developed countries [6]. Simple biscuits (without cream and ingredients such as peanuts, fruits, etc.) are ready to eat, affordable, and have a long shelf life and a wide range of tastes. They represent a self-sufficient part of the bakery industry [7] and are usually used for “snacks” by both children and adults [8]. Cookies (from soft dough), mainly containing flour, fat, and sugar, are popular bakery snacks due to their low cost, good taste, texture, and storage capacity [9]. The United States, India, and China are the biggest markets for cookies, while in Western Europe, Italy and Spain are the main demand markets [10]. Cookies are highly appreciated in the northeast of Romania (the Bucovina region), where there are small processing units for pastry products. Although cookies are commonly consumed in the rural areas of northern Romania, today, these products, whose sales are growing significantly, are becoming fashionable in urban cities. In bakery products, but especially in cookies, shortening and margarine are some of the most used fat sources that have a flexible base fat system that provides the desired sensory and technological qualities of a certain product. Thus, alternatives have been developed, considering polymers, polysaccharides, proteins, or lipids to replace saturated fats [11]. In the food industry, oleogels offer properties similar to solid fats with low levels of trans and saturated fat and can be carriers of bioactive compounds, maintaining desirable functional properties of fats, such as mouthfeel, creaminess, and stability [12]. Oleogels, characterized by their semisolid matrix, offer a promising alternative to trans and saturated fats in food products, which are known to be harmful to health, mimicking their texture and functionality [13]. Oleogels can protect bioactive compounds from degradation and improve the nutritional profile and texture of spreads, bakery, confectionery, dairy, and meat products by increasing the bioavailability of nutrients and reducing saturated fat content.

*Cannabis sativa* L. is an annual plant, which is widely spread around different geographical zones that produces small seeds. Cold pressing of these seeds (35% oil) allows obtaining hemp seed oil, which is mainly used as food [14,15]. Hemp seed oil (*Cannabis sativa* L.) is considered a healthy oil and contains a unique ω-6/ω-3 fatty acid ratio of 3:1, which can be optimal for human nutrition and, therefore, suitable for both human consumption and utilization in the pharmaceutical and cosmetic industries [16,17,18]. People from Mediterranean areas have low rates of coronary heart disease because the n-6/n-3 ratio of hemp seed oil is similar to the n-6/n-3 ratio found in their diets [19]. Cold-pressed hemp oil, a natural product, is an excellent source of protein, fatty acids, enzymes, biologically active compounds, and vitamins associated with hemp seed components [20]. The bioactive compounds, but also the unsaponifiable fraction, offer health benefits, such as anti-cancer and anti-cardiovascular diseases, antimicrobial, anti-inflammatory, anti-thrombotic, anti-arterogenic, and hypocholesterolemic [18,21].

In this work, cold-pressed hempseed oil oleogels were prepared with 9% natural waxes, including beeswax (BW), carnauba wax (CW), candelilla wax (DW), rice bran wax (RW), and three oleogelation agents, respectively, sitosterol (S), pea protein (PP), and xanthan gum (XG), to evaluate their potential as solid fat replacements in cookies. In previous studies, oleogels elaborated with several vegetable oils and waxes of different types were analyzed [13,22,23]. Considering the technological feasibility, oil binding capacities, and fatty acid composition, the use of hemp seed oil was chosen for the development of oleogels and the reformulation of the cookie recipe. The objective of the research was to analyze the effect of replacing saturated fats with new oleogels prepared with hemp seed oil on the textural and sensory properties of cookies.

## 2. Results and Discussion

### 2.1. Texture Profile Analysis of Dough and Cookies

The results of the cookie dough texture analysis are shown in Table 1. The hardness increased with the replacement of margarine by oleogels, with the highest increase (4052.09 ± 0.12) in sample S5_S_HO with sitosterol as an oleogelation agent. In the case of samples of dough with oleogels obtained from hemp oil and natural wax, an increase in hardness values was observed for all samples, and the highest values were recorded for the sample with rice bran wax (S4_RW_HO), followed by the sample with beeswax (S1_BW_HO). The increase in the hardness of cookie doughs with oleogels was also observed by Tanislav et al. [24]. The hardness of cookie doughs with oleogels is influenced by the hardness of the oleogels, as harder oleogels can adversely affect the processing of the dough. The addition of 9% wax resulted in oleogels with higher hardness and lower stickiness [23]. The increase in hardness and decrease in adhesiveness may be due to the higher level of saturated fatty acids in these types of oleogels. Fully saturated with hydrogen and showing a more rigid and fixed structure, these are more solid and compact in the dough system [23]. At the same time, the hardness of oleogels indicates that they may be an alternative to saturated fats [24]. Oleogels form strong interactions with interacting surfaces, thus promoting adhesion. Adhesivity is the force required to remove food adhering to a particular surface, and the negative values shown in Table 1 signified a downward-directed stress [25]. Oleogels form strong interactions with interacting surfaces, thus promoting adhesion according to Muskat et al. [26]. The dough of the cookie sample with oleogel from hemp oil and rice bran wax (S4_RW_HO) had the highest adhesion, followed by sample S5_S_HO (with sitosterol as an oleogelation agent). Adhesivity is influenced both by the type of the oleogelation agent but also by the type of oil used to obtain the oleogels added to cookies; for example, Manohar and Rao [27] showed that dough obtained with rapeseed oil oleogels and structuring agents (ethylcellulose, candelilla wax, rice bran wax, white beeswax, yellow beeswax, and monoacylglycerol) had greater adhesiveness compared to dough with palm oil.

The springiness index correlated with stickiness tells us the recovery properties of the dough (a value of 1 indicates a fully elastic material, and 0 indicates a fully viscous material) [28]. In the cookie manufacturing process, the samples containing oleogels with hemp oil (HO), rice bran wax (RW), and xanthan gum (XG) (S4_RW_HO, S7_XG_HO) behaved best during rolling and shaping operations, although the springiness index values are very close. The results obtained for the springiness index showed that the values are not statistically significant. Cohesiveness showed opposite trends, indicating that a more cohesive dough has a lower recovery capacity after being subjected to stress, thus increasing its value. Cohesiveness decreased for the dough samples with oleogels from natural wax, except for the sample with candelilla wax (S2_DW_HO), but the values are not statistically significantly different. Sample S7_XG_HO (with xanthan gum as an oleogelation agent) had the highest value (0.35 ± 0.13) for cohesiveness, but was also not statistically different from the values of the other samples. The decrease in cohesiveness values indicates the lowest internal resistance of the dough samples with oleogels [29]. The obtained values of gumminess varied significantly, which means that the overall effect of the energy required to break down the internal forces of the dough varies considerably [30]. Adding oleogels to foods can lead to a gummy texture [31]. The increased gumminess of cookie samples with oleogel (especially from pea protein and hemp seed oil) may be due to the ability of pea protein to bind water and fat [32]. According to Tanislav et al. [29], fat reduces the stickiness of the dough surface and also oleogels based on the results of the present study. The results obtained using Pearson correlation for dough textural parameters indicated that hardness showed negative correlations with adhesiveness (r = −0.379), springiness (r = −0.199), cohesiveness (r = −0.529), and stickiness and positive correlations with gumminess (r = 0.716), while adhesiveness showed a negative correlation with springiness (r = −0.206) and positive correlations with cohesiveness (r = 0.609), gumminess, and stickiness. Springiness had a negative correlation with gumminess (r = −0.076) and positive correlations with cohesiveness and stickiness. Cohesiveness had a positive correlation with gumminess and a negative correlation with stickiness, while gumminess had a negative correlation with stickiness.

Texture analysis of cookies was performed by measuring the hardness, strength, and fracturability of cookies (Table 2). The hardness increased significantly from 4409.83 ± 0.13 g (control sample) to 7085.33 ± 0.15 g in sample S5_S_HO, a cookie prepared with hemp oil sitosterol oleogel, whereas sample S2_DW_HO had the lowest hardness value of 4048.09± 0.14 g. These findings are similar to other research [33], in which the textural properties of cookies made with oleogels produced by combining soy wax and rice bran oil were evaluated. Pradhan et al. [33] reported hardness values between 4400 and 4800 g for oleogel and butter cookie samples. In another study, the hardness of cookies prepared with commercial corn oil oleogels and four kinds of emulsifiers was significantly higher than that of the shortening cookies [34]. Excessive hardness will reduce the texture of the cookies [35]; a higher hardness was obtained for cookie samples with xanthan gum and sitosterol oleogels (samples S7_XG_HO and S5_S_HO). A significantly higher hardness of sitosterol oleogels attributed to superior structuring ability was observed by Tanislav al. [24]. The type of oleogelling agents and the proportions of oleogel influence the hardness of the baked product [36]. Fracturability refers to how easily the cookies will break [24]. In the present study, the fracturability values varied from 4.27 ± 0.13 mm (sample S6_PP_HO) to 14.99 ± 0.11 mm (sample S7_XG_HO). The cookie sample S7_XG_HO, with hemp oil oleogel and xanthan gum, had a higher fracturability value than the control sample, while the other samples had lower values compared to the control sample.

### 2.2. Rheological Characterisation of Dough with Oleogels

**Figure 1:**
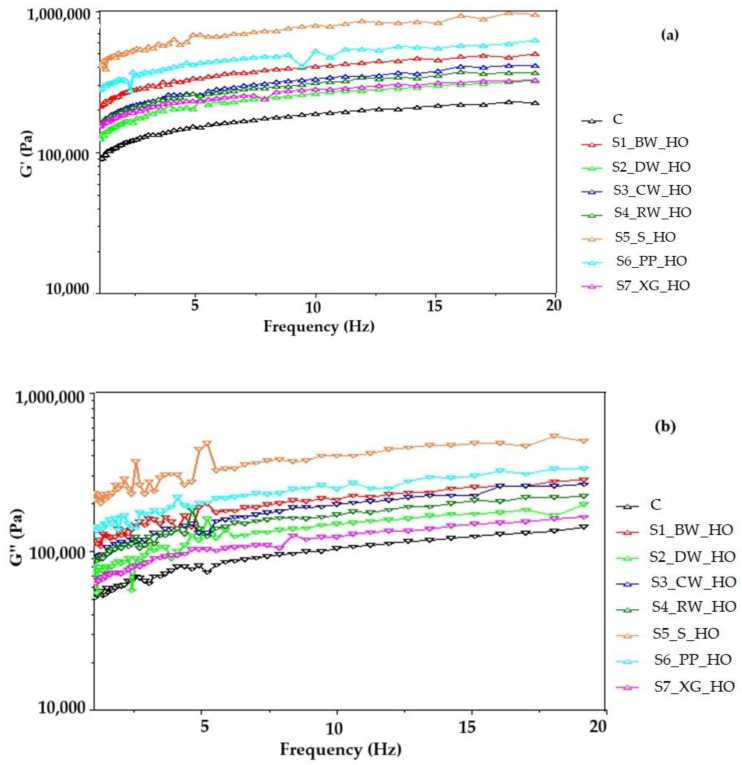
Variation of the modulus of (**a**) elasticity (G′) and modulus of (**b**) viscosity (G″) with the angular frequency for hemp oil (HO) oleogel dough samples.

The viscoelastic properties of the soft dough measured as a function of frequency are shown in Figure 1a,b.

**Figure 2:**
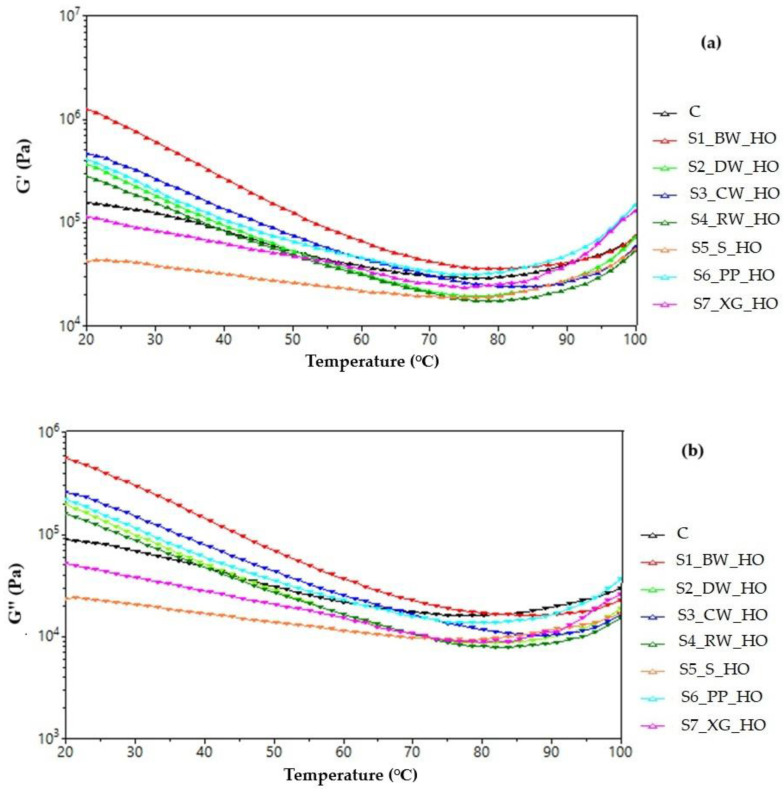
Variation of the modulus of (**a**) elasticity (G′) and modulus of (**b**) viscosity (G″) with the temperature for hemp oil (HO) oleogel dough samples.

Rheological measurements indicated that all the dough samples had an elastic solid type of behavior, and they presented higher values of G′ (storage mode) than G″ (loss mode) in the frequency range tested. The addition of oleogel increased the storage (G′) and loss moduli (G″) of the cookie dough compared to the control sample, indicating a more elastic and viscous character and thus stiffer composite dough networks. The higher lipid content of the oleogels contributes to the higher temporal stability of the dough network [37]. The cookies prepared in this study were classified as a soft dough product; under dough mixing conditions, the rheological properties of oleogels change, and they can quickly lose their solid structure as a result of shear forces and become much softer than shortening [38]. Similarly, variations in the modulus of elasticity (G′) and modulus of viscosity (G″) with temperature are shown in Figure 2a,b. As the temperature increased, the values of G′ and G″ decreased slightly, possibly due to hydrolysis of damaged starch by amylase according to Tang and Liu [39]. By analyzing the changes in the G′ and G″ modulus values as a function of temperature, it was found that there was a common trend of progressive decrease until a certain temperature was reached, which indicated a decrease in interactions in the system [40]. The doughs with oleogel obtained with xanthan gum and the doughs with sitosterol show low viscoelastic behavior. Xanthan gum did not produce high elasticity for short sugar cookie doughs, and the same behavior was observed for the doughs with sitosterol. The cookies obtained from these doughs showed the highest hardness values, and the maximum force point was the highest for the cookies with gum and those with sitosterol.

### 2.3. Cookies’ Color

Maillard and caramelization reactions are responsible for the coloring and flavor of cookies [41]. There are significant differences between the color of the control sample and the color of cookies with oleogels (Table 3). The lightness (L*) values of the cookie samples with oleogels decreased compared to the control sample. According to Sobolev et al. [42], in order not to interfere with food color perception, a high value of L* is preferable. In the case of cookies with oleogel from hemp oil and natural wax, the highest L* value was for the cookie sample with beeswax oleogel (59.94 ± 0.13), while for the cookie samples with oleogelation agents, the cookie sample with oleogel from hemp oil and xanthan gum had the highest value (63.36 ± 0.38). It was observed that the cookie samples with oleogels from hemp oil and natural waxes are darker than cookie samples with oleogels from hemp oil and oleogelation agents, like xanthan gum and pea protein. Pang et al. [35] reported a decrease in the L* values of the oleogel cakes from rice bran oil (RBO) with the following oleogelling agents: candelilla wax (CDW), beeswax (BW), rice bran wax (RBW), and carnauba wax (CRW) [30]. Also, Zulfiqar et al. [28] indicated that the L* value decreased with an increasing percentage of using a blend of corn oil and sunflower oil oleogel with beeswax as an oleogelator in cookies. The a* values of the oleogel cookie samples decreased compared to the control sample, with the exception of the hemp oil and pea protein oleogel cookie sample (S6_PP_HO). All values were positive, indicating that the red tone dominates the green tone in all samples of oleogel-based cookies. Also, positive values were obtained for the b* color parameter for all cookie samples, indicating that the yellow tone dominates the blue tone. Cookies formulated with oleogel can be characterized by a shade of red and yellow according to Tanislav et al. [29]. The results obtained for total color difference (ΔE) indicate major color differences between the oleogel-based cookies and the control sample. The higher color differences were observed for oleogel-based cookies with hemp oil and candelilla wax (S2_DW_HO) and with rice wax (S4_RW_HO) and for cookie samples with sitosterol used as an oleogelation agent (S5_S_HO). The differences between the L* color parameter values of margarine and margarine cookie samples were about 6%, with the L* values decreasing in the margarine cookie sample (C), which meant that the control sample was darker in color than the margarine. The L* values of S6_PP_HO, S1_BW_HO, and S5_S_HO cookie samples increased by 73.25%, 50.63%, and 27.62% compared to the L* values of PP, BW, and P with HO oleogel samples. The PP_HO, BW_HO, and S_HO oleogel samples are the darkest in color among all the investigated oleogel samples, and the addition of oleogels to the cookie samples leads to the opening of their color. For the S2_DW_HO sample, no color changes were observed regarding the L* parameter, while for the S3_CW_HO sample, a decrease in the L* values of approximately 2% was determined compared to the oleogel sample. If in the case of the S4_RW_HO cookie sample the L* values increased by about 15% compared to the L* value for the oleogels obtained from RW and HO, in the case of the S7_XG_HO cookie sample, the color parameter L* values decreased by 16%. The oleogel sample with XG and HO is the lightest in color among the oleogel samples, but the addition of the oleogel in the cookies leads to the darkening of their color. All the oleogel samples, as well as the margarine sample, had negative values for the color parameter a*, indicating green-colored samples, while all the cookie samples had positive values for this parameter, indicating reddish-colored samples. For all samples (margarine, oleogels, oleogel cookies), positive values for the color parameter b* were determined, indicating a yellow color of the samples. The addition of oleogels in cookies from BW, DW, RW, and XG with HO led to an increase in the values of the color parameter b*, while the addition of oleogels from CW, S, and PP with HO led to a decrease in the values of this parameter compared to the values obtained for the respective oleogels.

### 2.4. Sensory Evaluation

**Figure 3:**
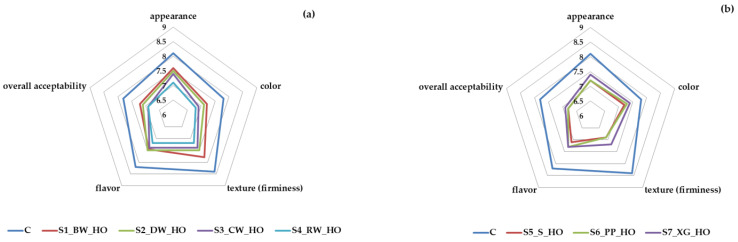
Sensory evaluation of cookies with hemp seed oil oleogels: (**a**) natural waxes and (**b**) other oleogelation agents.

Sensory attributes for cookies with oleogels from hemp seed oil and natural waxes (beeswax, carnauba wax, candelilla wax, rice bran wax) and other oleogelation agents (sitosterol, pea protein, and xanthan gum) are illustrated in Figure 3. Consumers scored the appearance, color, texture, flavor, and overall acceptability of the eight cookie samples presented with number codes. The group determined the sensory attribute “flavor”, which defines the overall perception of the aroma and taste associated with cookies after they are chewed and swallowed, as indicated by Yılmaz and Öğütcü [43]. The control cookie sample (with margarine) scored highest for all sensory attributes. The cookie sample with oleogel from hemp seed oil and beeswax (S1_BW_HO) was more appreciated for appearance and texture, while the cookie sample with oleogel from hemp seed oil and candelilla wax (S2_DW_HO) and the cookie sample with oleogel from hemp seed oil and xanthan gum (S7_XG_HO) were more appreciated for flavor and color, respectively. The least appreciated samples were S4_RW_HO for appearance and color, S6_9PP_HO for texture, and S5_9S_HO for flavor. Regarding the overall acceptability, the highest scores for cookies with oleogels were obtained by cookie samples S1_BW_HO and S2_DW_HO, while the lowest score was obtained by cookie sample S6_9PP_HO. The color of cookies with hemp seed oleogels was darker than that of control cookies. Similar results were reported by Flores-García et al. [44] using organic candelilla wax and extra-virgin linseed oil. The familiar golden-brown color that the cookie samples exhibited may be one of the reasons why evaluators liked these samples; the dark-brown colors resulting from the intense Maillard reaction may appeal much less to consumers. Evaluators downgraded the aroma and taste of the products due to the hemp oil having a slightly bitter taste and specific smell. With a somewhat bitter aftertaste, hemp oil can be used as a substitute for olive oil because it has a color similar to that of olive oil [45]. The oxidation of fats from cookies may influence consumer acceptability. It was observed that the oleogel’s oxidative stability increased with the addition of different types of wax [13]. The peroxide value for hemp seed oil can be between 2.18 and 7.73 meqO_2_/kg, and the addition of candelilla wax (9%) can decrease the peroxide value up to 1.25 meqO_2_/kg [46]. β-sitosterol exhibits antioxidant properties and acts as a free radical scavenger [47]. β-sitosterol donates hydrogen atoms faster to free radicals and reduces the oxidation of polyunsaturated fatty acids. β-sitosterol in higher concentrations could not only reduce lipid oxidation but also form a stronger gel [48]. Panagiotopoulou et al. reported similar results for lipid oxidation in frankfurters prepared with phytosterol-based organogels [49].

### 2.5. Principal Component Analysis

**Figure 4:**
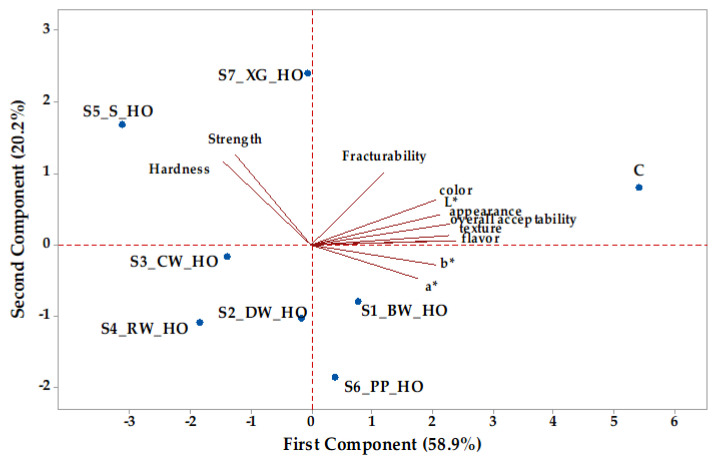
Principal component analysis biplot depicting the relationship between parameters of cookie samples (HO—hemp seed oil, oleogelling agents: BW—beeswax; DW—candelilla wax; CW—carnauba wax; RW—rice bran wax; S—sitosterol; PP—pea protein; XG—xanthan gum; L*, a*, and b*—color parameters).

The relationship between textural and color parameters of cookie samples with oleogels is shown in Figure 4. Principal component 1 (PC1) accounted for 58.9% of the total variation, while PC2 accounted for 20.2% of the total variation. All investigated parameters had positive loadings on PC1, except for hardness (−0.224) and strength (−0.194), which had negative loadings on PC1. Also, all investigated parameters had positive loadings on PC2, except for color parameters a* (−0.211) and b* (−0.122). Small angles between flavor, texture, appearance, overall acceptability, L*, and color reflected a great degree of correlation between these variables. These attributes were dominant in the control cookie sample, while strength and hardness predominated in cookies with oleogels from hemp seeds oil with xanthan gum and sitosterol. Additionally, color parameters, such as a* and b*, were key parameters in cookie samples with oleogels from hemp seed oil and oleogelling agents, like beeswax and pea protein. The control sample (C) had positive values for both PC1 and PC2, while samples S1_BW_HO and S6_PP_HO (situated to the right in the score biplot according to Figure 4) had positive values for PC1 and negative values for PC2. The other cookie samples are situated to the left in the score biplot. Three samples (S2_DW_HO, S3_CW_HO, and S4_RW_HO) had negative values for PC1 and PC2, and the other two samples (S5_S_HO, S7_XG_HO) had negative values for PC1 and positive values for PC2.

## 3. Conclusions

The possibility of using oleogels, obtained from hemp seed oil and different oleogeling agents in cookie production, was investigated. This study showed that the type of oleogel used, as a margarine substitute, affects the textural parameters (adhesiveness, springiness, cohesiveness, and stickiness) of the obtained cookie dough. Replacing fat with oleogels led to an increase in the hardness of cookie doughs, and also, the addition of oleogel increased the viscoelastic moduli of the cookie doughs. Cookies with oleogels are darker in color, with a red and yellow hue. The sensory results indicate that consumers may choose oleogel cookies (made from hemp oil and wax (especially beeswax and candelilla), which are more popular than the others). Hemp seed oleogels with different gelators have practical solid fat replacement applications and present possible opportunities to formulate healthier cookies.

## 4. Materials and Methods

### 4.1. Materials

Hemp seed oil (HO) was purchased from a grocery store (Suceava, Romania), while candelilla wax (DW) and carnauba wax (CW) were supplied by Sigma-Aldrich (Hamburg, Germany). Beeswax (BW) and rice bran wax (RW) were obtained from Merck, Bucharest, Romania, while sitosterol (S), pea protein (PP), and xanthan gum (XG) were purchased from a health food store (Suceava, Romania). Wheat flour (type 480), sugar, milk powder, eggs, salt, and sodium bicarbonate were purchased from a local supermarket (Suceava, Romania).

### 4.2. Oleogel Preparation

Oleogels were obtained, according to the method described by Ropciuc et al. [13], using hemp seed oil and oleogelation agents (beeswax, candelilla wax, carnauba wax, sitosterol, pea protein, xanthan gum) in the percentage of 9%. A hot plate with an orbital stirrer was used to heat the oil and wax to 80 °C until the wax was completely dissolved. A mixture was obtained and poured into tubes for solidification and was then stored under refrigerated conditions at 4 °C for five days until analysis [23]. In the case of pea protein and xanthan gum, stable foams were first prepared according to Mohanan et al. [50] and Mohanan et al. [51]. Afterwards, oleogels were prepared by adding a hot mixture (80 °C) of hemp seed oil to freeze-dried foams.

### 4.3. Sample Preparation

Different doughs were prepared starting from wheat flour type 480 to obtain a final quantity of about 200 g. The ingredients were cookie mixture (105 g wheat flour, 30 g sugar, 10.5 g milk powder, 33.6 g mixing eggs, 0.6 g salt, 0.3 g sodium bicarbonate), oleogel, and margarine (for the control sample), and they were added as indicated in the experimental design model (Table 4). The dough samples have been formulated so that the fat content is the same in all samples. After baking and cooling, the fat content is not changed; only the water evaporates from the product.

**Figure 5:**
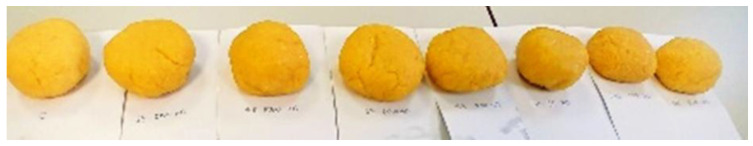
Visual appearance of dough samples.

The used sample codes were as follows: the control sample (C) with vegetable shortening and cookie samples with 10% oleogel: S1_BW_HO (oleogel from hemp seed oil and 9% beeswax), S2_DW_HO (oleogel from hemp seed oil and 9% candelilla wax), S3_CW_HO (oleogel from hemp seed oil and 9% carnauba wax), S4_RW_HO (oleogel from hemp seed oil and 9% rice bran wax), S5_S_HO (oleogel from hemp seed oil and 9% sitosterol), S6_PP_HO (oleogel from hemp seed oil and 9% pea protein), and S7_XG_HO (oleogel from hemp seed oil and 9% xanthan gum). All ingredients were homogenized in a bowl with a kitchen mixer (KitchenAid Professional Mixer, Greenville, OH, USA) for 13 min at speed 4. The cookie dough was spread to a thickness of 8 mm, and 5 cm diameter discs were cut using a circular mold. The cookies were placed on a tray and baked in an electric oven at 200 °C for 25 min (Figure 5). After cooling to room temperature, the cookie samples were packed in polypropylene bags and stored at room temperature until analysis.

### 4.4. Texture Profile Analysis of Dough and Cookies

After kneading, the dough was allowed to stand for 12 h under refrigerated conditions. The textural properties of the dough were determined at 20–22 °C. The textural properties of the dough and cookies were measured with the Perten TVT 6700 texturometer (Perten Instruments, Hägersten, Sweden). The textural properties were determined as described by Leahu et al. [52] with minor modifications. To determine the textural characteristics, the compression test was applied using 45 mm Diameter, Stainless Steel (P-CY45S), the compression percentage per sample was 30%, and the compression force was 10 g. The characteristics of dough hardness, stickiness, cohesiveness, elasticity, adhesiveness, and gumminess were analyzed in triplicate. Cookie samples were placed on the cutting table 6 h after baking and cooling. Cutting samples were performed on 1 cm high biscuits, previously measured with a cookie cutter. A Craft Blade Knife (P-CBK) with a cutting force of 25 g was used. The maximum point of force was noted, which expresses the breaking point of the biscuit. The test carried out for the cookies was one of fracturability (a measure of the tendency to fracture, in response to force). The sample was placed on a support with 2 parallel plates separated by 20 mm. The knife descended on the sample until it fractured. The initial descent speed of the cylindrical part was 1 mm/s, and the speed during the test was 3 mm/s. The force applied to the samples was 50 g. The blade cuts through the cookies, and the force required to do so is recorded as cookie strength [gmm]. Three repeated measurements were performed on each sample.

### 4.5. Rheological Analysis of Dough

The dynamic rheometer, model Haake Mars 40 (Thermo Fisher Scientific, Karlsruhe, Germany), was used to determine dough viscosity. Viscosity as a function of time (10 min) at a constant shear rate of 100 s^−1^ and a frequency of 1–100 Hz and graphical representations of viscosity curves were determined using Haake Rheo Win Data Manager 4 Software for Haake Mars 40 Rheometer (Thermo Fisher Scientific, Karlsruhe, Germany) according to the method previously described by Ropciuc et al. [23]. Dough samples were kept in plastic bags to prevent drying. The viscoelastic properties of the dough were determined at varying frequency and temperature. Plate/plate geometry (40/80 mm) with a 3 mm gap was used. The viscoelastic modulus was determined at a frequency of 1–20 Hz and a temperature of 20 °C. In order to know the baking behavior of dough with different oleogelating agents, the behavior of the viscoelastic modulus at variable temperatures was determined. The viscoelastic and elastic properties of biscuit dough at variable temperatures between 20 and 90 °C were determined.

### 4.6. Determination of Color Parameters

Color parameters were analyzed using a Minolta Chroma Meter (Model CR 310, Minolta Camera Co. Ltd., Tokyo, Japan). The results were expressed in terms of L* (lightness/darkness), a* (redness/greenness), and b* (yellowness/blueness) using the average of 3 readings per sample on different points of the cookie’s surface [21]. Total color difference (*ΔE*) was calculated by the following equation [41]:

(1) $$\Delta E=\sqrt{{\left(L^{*}-L_{0}^{*}\right)}^{2}+{\left(a^{*}-a_{0}^{*}\right)}^{2}+{\left(b^{*}-b_{0}^{*}\right)}^{2}}$$

where L*, a*, and b* are color parameters of cookies with oleogels, while L_0_*, a_0_*, and b_0_* are color parameters of control cookies. According to Xiong et al. [41], if *ΔE* < 1, there are no differences between the samples; 1 < *ΔE* < 3 indicates minor color differences, and *ΔE* > 3 indicates major color differences.

### 4.7. Determination of the Cookies’ Sensory Attributes

The cookie samples were proposed for sensory analysis to a group of 45 members selected from the students of the Faculty of Food Engineering using a defined sensory language (taste, flavor, texture, color, and overall acceptability) on a 9-point hedonic scale, where 9 was extremely like and 1 was extremely dislike, to understand consumer acceptance [53]. In hedonic tests, untrained consumers are used as testers. The coded samples were offered to the tasters, and the sensory evaluation was performed by using the preference test. The preference or ranking test was carried out according to the ISO 8587:2006 standard [54].

### 4.8. Statistical Analysis

Minitab 17 software (Minitab, Inc., State College, PA, USA) was used for statistical analysis. One-way analysis of variance (ANOVA) with a 95% confidence interval (*p* < 0.05) and Tukey’s test were considered to compare the results obtained; also, principal component analysis and Pearson correlation were performed. ANOVA shows whether there is a statistically significant difference between the means of three or more groups of data (when the null hypothesis is rejected, *p* ≤ 0.05), while Tukey’s test (one of the post hoc tests) indicates which of these are different. Principal component analysis (PCA) was conducted to assess the relationship between textural, color, and sensory attributes and to evaluate similarities and differences between cookie samples.

## Figures and Tables

**Table 1:** Texture properties of the cookie dough with oleogels.

Sample	Hardness (g)	Adhesiveness (J)	Springiness (mm)	Cohesiveness	Gumminess	Stickiness
C	1589.99 ± 0.19 ^h^	−50.43 ± 0.13 ^b^	1.00 ± 0.11 ^a^	0.27 ± 0.13 ^a^	423.44 ± 0.14 ^f^	−9.47 ± 0.16 ^a^
S1_BW_HO	2146.78 ± 0.18 ^d^	−450.38 ± 0.18 ^f^	1.00 ± 0.19 ^a^	0.18 ± 0.12 ^a^	384.73 ± 0.13 ^g^	−67.72 ± 0.11 ^f^
S2_DW_HO	1598.77 ± 0.17 ^g^	9.00 ± 0.12 ^a^	0.99 ± 0.19 ^a^	0.32 ± 0.11 ^a^	503.65 ±0.14 ^e^	−83.75 ± 0.14 ^g^
S3_CW_HO	1981.84 ± 0.14 ^e^	−259.66 ± 0.16 ^e^	1.00 ± 0.18 ^a^	0.19 ± 0.12 ^a^	384.91 ±0.03 ^g^	−51.45 ± 0.15 ^b^
S4_RW_HO	2343.85 ± 0.15 ^c^	−856.27 ± 0.17 ^h^	1.00 ± 0.12 ^a^	0.22 ± 0.13 ^a^	507.00 ± 0.19 ^d^	−57.51 ± 0.11 ^c^
S5_S_HO	4052.09 ± 0.12 ^a^	−466.66 ±0.16 ^g^	0.99 ± 0.14 ^a^	0.18 ± 0.13 ^a^	734.47 ± 0.17 ^b^	−63.29 ± 0.19 ^d^
S6_PP_HO	2998.8 ± 0.18 ^b^	−115.71 ±0.11 ^c^	1.00 ± 0.19 ^a^	0.26 ± 0.11 ^a^	792.04 ± 0.04 ^a^	−66.25 ± 0.14 ^e^
S7_XG_HO	1711.82 ± 0.12 ^f^	−185.41 ± 0.14 ^d^	1.00 ± 0.07 ^a^	0.35 ± 0.13 ^a^	601.30 ± 0.13 ^c^	−94.73 ± 0.13 ^h^

Values are mean ± standard deviation (*n* = 3). Different superscript letters (a–h) in the same column indicate significant differences between values at the *p* < 0.05 level.

**Table 2:** Texture parameters of the cookies with oleogels.

Sample	Hardness (g)	Strength (g/mm)	Fracturability (mm)
C	4409.83 ± 0.13 ^e^	220.49 ± 0.13 ^e^	11.42 ± 0.10 ^b^
S1_BW_HO	4387.91 ± 0.17 ^f^	219.39 ± 0.15 ^f^	5.28 ± 0.13 ^e^
S2_DW_HO	4048.09 ± 0.14 ^h^	202.40 ± 0.14 ^h^	7.41 ± 0.09 ^c^
S3_CW_HO	5284.50 ± 0.10 ^d^	264.22 ± 0.17 ^c^	6.03 ± 0.14 ^d^
S4_RW_HO	5287.50 ± 0.15 ^c^	226.68 ± 0.11 ^d^	5.59 ± 0.12 ^e^
S5_S_HO	7085.33 ± 0.15 ^a^	354.26 ± 0.13 ^a^	4.77 ± 0.15 ^f^
S6_PP_HO	4074.84 ± 0.17 ^g^	203.74 ± 0.18 ^g^	4.27 ± 0.13 ^g^
S7_XG_HO	6383.59 ± 0.12 ^b^	319.17 ± 0.16 ^b^	14.99 ± 0.11 ^a^

Values are mean ± standard deviation (*n* = 3). Different superscript letters (a–h) in the same column indicate significant differences between values at the *p* < 0.05 level.

**Table 3:** Color parameters of the cookie samples.

Samples	L*	a*	b*	ΔE
C	68.31 ± 1.52 ^a^	3.35 ± 0.21 ^b^	36.25 ± 1.16 ^a^	-
S1_BW_HO	59.94 ± 0.13 ^c^	2.23 ± 0.05 ^c^	31.96 ± 0.21 ^bc^	9.55 ± 1.05 ^bc^
S2_DW_HO	53.63 ± 0.09 ^d^	1.21 ± 0.03 ^d^	31.57 ± 0.67 ^c^	15.55 ± 1.40 ^a^
S3_CW_HO	55.66 ± 0.69 ^d^	1.31 ± 0.03 ^d^	30.42 ± 0.24 ^cd^	14.14 ± 1.62 ^ab^
S4_RW_HO	53.88 ± 1.64 ^d^	1.98 ± 0.09 ^c^	31.71 ± 0.39 ^c^	15.23 ± 2.80 ^a^
S5_S_HO	55.12 ± 0.82 ^d^	0.89 ± 0.04 ^e^	28.86 ± 0.65 ^d^	15.36 ± 1.78 ^a^
S6_PP_HO	62.44 ± 0.42 ^bc^	3.87± 0.05 ^a^	36.02 ± 0.07 ^a^	5.96 ± 1.92 ^c^
S7_XG_HO	63.36 ± 0.38 ^b^	2.03 ± 0.04 ^c^	33.41 ± 0.30 ^b^	6.02 ± 1.15 ^c^

Values are mean ± standard deviation (*n* = 3). Different superscript letters (a–e) in the same column indicate significant differences between values at the *p* < 0.05 level.

**Table 4:** Cookie dough recipe formulations.

Samples	Cookie Mixture ^1^	Margarine, g	Oleogel, g
C	180	20	-
S1_BW_HO	180	-	20
S2_DW_HO	180	-	20
S3_CW_HO	180	-	20
S4_RW_HO	180	-	20
S5_S_HO	180	-	20
S6_PP_HO	180	-	20
S7_XG_HO	180	-	20

^1^ Cookie mixture: 105 g wheat flour, 30 g sugar, 10.5 g milk powder, 33.6 g mixing eggs, 0.6 g salt, 0.3 g sodium bicarbonate. HO—hemp seed oil; oleogelling agents: BW—beeswax; DW—candelilla wax; CW—carnauba wax; RW—rice bran wax; S—sitosterol; PP—pea protein; XG—xanthan gum.

## Data Availability

The original contributions presented in the study are included in the article; further inquiries can be directed to the corresponding author.
